# Spinning straw into gold: description of a disruptive rheumatology research platform inspired by the COVID-19 pandemic

**DOI:** 10.1186/s13075-021-02574-z

**Published:** 2021-08-05

**Authors:** L. Boekel, F. Hooijberg, E. H. Vogelzang, P. L. Klarenbeek, W. H. Bos, S. W. Tas, G. J. Wolbink

**Affiliations:** 1 Department of Rheumatology, Amsterdam Rheumatology and Immunology Center, location Reade, Dr. Jan van Breemenstraat 2, 1056 AB Amsterdam, the Netherlands; 2 Department of Medical Microbiology and Infection Prevention, Amsterdam UMC, location AMC, 1105 AZ Amsterdam, the Netherlands; 3 Amsterdam Rheumatology and Immunology Center, Amsterdam UMC, location AMC, Department of Rheumatology and Clinical Immunology, Meibergdreef 9, 1105 AZ Amsterdam, the Netherlands; 4 Department of Immunopathology, Sanquin Research and Landsteiner Laboratory Academic Medical Center, Plesmanlaan 125, 1066 CX Amsterdam, the Netherlands

## Abstract

Clinical research projects often use traditional methods in which data collection and signing informed consent forms rely on patients’ visits to the research institutes. However, during challenging times when the medical community is in dire need of information, such as the current COVID-19 pandemic, it becomes more urgent to use digital platforms that can rapidly collect data on large numbers of patients. In the current manuscript, we describe a novel digital rheumatology research platform, consisting of almost 5000 patients with autoimmune diseases and healthy controls, that was set up rapidly during the COVID-19 pandemic, but which is sustainable for the future. Using this platform, uniform patient data can be collected via questionnaires and stored in a single database readily available for analysis. In addition, the platform facilitates two-way communication between patients and researchers, so patients become true research partners. Furthermore, blood collection via a finger prick for routine and specific laboratory measurements has been implemented in this large cohort of patients, which may not only be applicable for research settings but also for clinical care. Finally, we discuss the challenges and potential future applications of our platform, including supplying tailored information to selected patient groups and facilitation of patient recruitment for clinical trials.

**Supplementary Information:**

The online version contains supplementary material available at 10.1186/s13075-021-02574-z.

## Introduction

Since the start of the COVID-19 pandemic in December 2019, countries around the world have implemented social distancing measures to reduce the spread of SARS-CoV-2. In healthcare, these measures have led to procrastination of many plannable interventions and an extensive reduction in outpatient visits. Consequently, clinical research projects using traditional methods, in which data collection and signing informed consent forms rely on patients’ visits to the research institutes, have been seriously impeded. However, the COVID-19 pandemic has also provided unique opportunities to answer important research questions regarding infection risks of vulnerable patients, such as patients with rheumatic diseases for whom prospective data are still scarce. Digital data collection is one of the few options left to set up prospective studies like this during the current COVID-19 pandemic. Therefore, the rheumatology community developed a digital research platform that rapidly collected enormous amounts of data on COVID-19 cases around the world [1]. In addition, large-scale cross-sectional studies collected data on vulnerable patient groups via digital surveys. In Denmark for example, a readily available nationwide registry for rheumatic patients (DANBIO) was used to send surveys to all registered patients, which resulted in thousands of responses in a short amount of time [2]. The above-mentioned initiatives demonstrate that a digitalized research environment facilitates (international) collaboration between research institutes and accelerates data collection and processing. Despite these major advantages, (prospective) cohort studies collecting data without requiring visits of participants to local research institutes are still scarce, since most studies do not only rely on (digital) questionnaires, but also on physical examination or laboratory tests. For laboratory tests, this can be overcome by the development of tools that facilitate collection of serological data without a visit to the clinic. In this paper, we describe how we developed a digital research platform and implemented serum collection via a finger prick for a large (still ongoing) prospective cohort study in patients with autoimmune diseases and healthy controls. In addition, we will discuss the advantages, challenges, and potential future applications of our platform.

## Study design

In April 2020, we set-up a prospective cohort study (Netherlands Trial Register, trial ID NL8513, 13-04-2020) to compare disease severity of COVID-19 between patients with autoimmune diseases and healthy controls. All adult patients with systemic autoimmune diseases from Reade and selected patient groups from the Amsterdam UMC locations AMC and VUmc (working together in the Amsterdam Rheumatology & Immunology Center) were invited via e-mail to participate in the study. Patients were asked, but not obliged, to register their own control subject who was of the same sex and similar age (< 5 years difference).

**Fig. 1:**
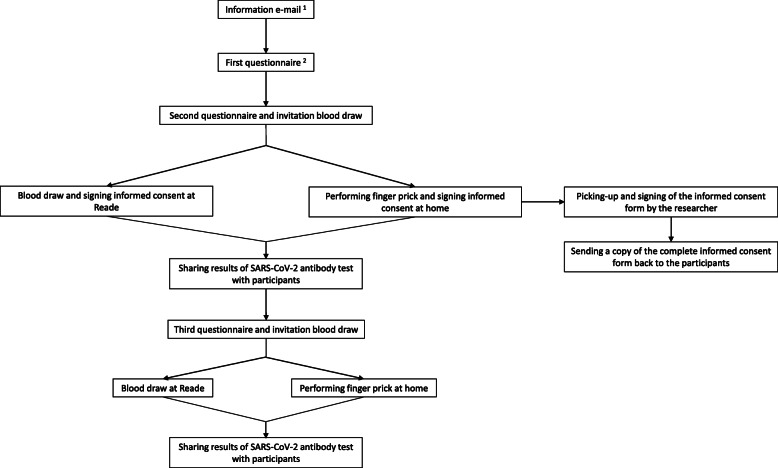
Flow chart of study procedures. Superscript digit 1 indicates the following: if patients did not want to participate in the study, they had to actively sign out. Otherwise, they would receive an invitation (and later reminders) for the first questionnaire. Superscript digit 2 indicates the following: it was explained that filling in the questionnaire meant giving permission to use the data for the study. Patients could also sign-up their own control subject in the first questionnaire

An overview of all study procedures is shown in Fig. 1. Clinical data were collected using digital questionnaires through a secure web portal developed by Brightfish at baseline and after 1–5 and 6–10 months of follow-up. Upon invitation per e-mail, every participant could create a personal account for the web portal using two-factor authentication. In case participants preferred not to create a personal account, the questionnaire could also be opened directly via a link attached in the e-mail. The questionnaires could be completed from any device, and data were stored in an encrypted database. Planned and pre-defined exports of the data were stored in the Castor cloud-based clinical data management system. Castor was also used to send the questionnaire to participants who experienced difficulties in completing the questionnaire via the web portal. A study code was assigned to all participants to pseudonymize the data. Both the web portal and Castor are compliant with the European Union’s General Data Protection Regulation (GDPR).

Serum samples were collected twice; between July and November, 2020, and between December 2020 and March, 2021. Participants could choose between visiting the outpatient clinic of Reade for regular blood withdrawal or using a finger prick set that would be send to their home addresses. A finger prick set contained instructions on performing the finger prick procedure, two copies of informed consent forms, materials to perform the finger prick, an anonymized coded tube to collect the blood and a return envelope. The combined costs of the materials and delivery of a single finger prick set were approximately 10 euros. Serum samples were analyzed for the presence of SARS-CoV-2-specific antibodies with a RBD-Ab bridging ELISA developed by Sanquin [3].

The main research team consisted of one full-time PhD student, 3 PhD students who supported the project at peak times (all from Reade), one principal investigator per investigational site, and several students and nurses who facilitated patient-recruitment within the other participating centers (Amsterdam UMC, locations VUmc and AMC). The web-based portal was developed by an ICT-expert of Brightfish, while the Castor database was built by our own study team. A team of technicians from Sanquin arranged the receival of the returned finger prick packages and analyzed all serum samples. The team of PhD students was responsible for communication with participants from all participating centers (via e-mail and telephone) and all other parties involved. Important intellectual input was acquired via close collaboration with experts in the field of immunology and rheumatology, both physicians and scientists.

## Scale and representativeness of the study

**Fig. 2:**
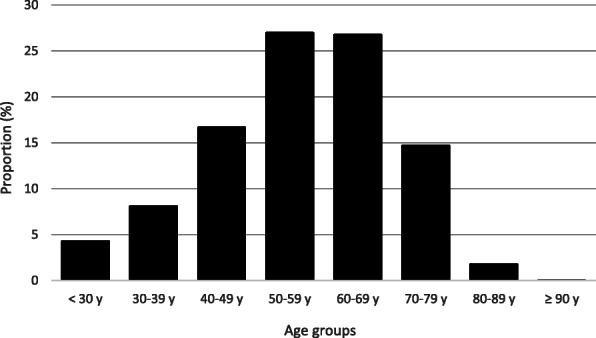
Age distribution of the study population

Between April and December, 2020, 4225 people were included in the study. Baseline characteristics of patients and are listed in Table 1. So far, serum samples have been collected from 3175 subjects. Blood was obtained via a finger prick by 81% of these subjects (*n* = 2575), and 34% (*n* = 1077) combined the blood withdrawal for the study with a regular physical appointment in the outpatient clinic. The mean age of the study population was 56 (SD 14) years, and the majority was female, 64% (*n* = 2780). In Fig. 2, the age distribution the study population is demonstrated. It can be seen that the elderly population is also well represented; 17% (*n* = 708) of all subjects were ≥ 70 years, and 2% (*n* = 82) were ≥ 80 years. These data thus indicate that not just the young but also older generations are capable of participating in our digital research platform.

**Table 1:** Baseline characteristics compared to healthy controls

Patient characteristics	Patients (*n* = 3128)	Controls (*n* = 1097)
Mean age—year	57 ± 14	55 ± 13
Female sex—no. (%)	1994 (64)	754 (69)
Mean BMI	26 ± 5	25 ± 4
Educational level*—no. (%)		
High	1155 (46)	530 (58)
Middle	783 (31)	244 (27)
Low	566 (18)	138 (15)
**Autoimmune disease type—no. (%)**		
Rheumatoid arthritis	1544 (49)	N.A.
Psoriatic arthritis	444 (14)	N.A.
Ankylosing spondylitis	423 (14)	N.A.
Axial or peripheral spondyloarthritis	38 (1)	N.A.
Juvenile idiopathic arthritis	47 (2)	N.A.
Systemic lupus erythematodes	159 (5)	N.A.
Vasculitis	44 (1)	N.A.
Polymyalgia rheumatica	99 (3)	N.A.
Sjogren’s disease	141 (5)	N.A.

Values are displayed as mean ± standard deviation (SD) or frequencies with corresponding valid percentages (%). BMI, body mass index. *Classification is based on the international standard classification of education (ISCED)

## Advantages

The rheumatology research platform described here has several advantages compared to traditional clinical research methods in large patient groups. First, the platform considerably accelerates the process of generating data that might be of interest for publication, which is important in challenging times when the medical community is in dire need of information. Because study invites and data collection both occur digitally, data from thousands of patients with autoimmune diseases and healthy controls were collected with relative ease and in a short amount of time. In addition, extensive manual data entry and processing by researchers were no longer necessary, as data were automatically processed into a database in the right format for analyses. Because of this, we were able to demonstrate already in the first few months of the COVID-19 pandemic that patients with autoimmune diseases adhered to isolation measures more strictly compared to the general population [4]. These results were submitted three months after approval of the study protocol by the medical ethical committee and published 1 month later in The Lancet Rheumatology. Second, our study design is very adaptable to unexpected developments or newly gained insights into study related topics, which is especially useful in times such as the COVID-19 pandemic when new relevant research questions rapidly succeed each other. For example, when vaccinations against COVID-19 were approved for public use, the perspective of patients with autoimmune diseases towards vaccination became relevant. We therefore added questions to one of our surveys with the aim to compare vaccination willingness among patients with autoimmune diseases to healthy controls and to explore underlying reasons for vaccine hesitancy. The results of these additional questions were submitted approximately one month and published within 3 months after they were first sent out to participants [5], which emphasizes the adaptive power and efficiency of our platform. Third, our study platform facilitates multicenter research because it is easily scalable and institute overarching. In general, hospitals use different electronic health record systems that may not have the ability to send out digital surveys, let alone in the same format. Creating a platform that ensures uniform data collection in a single database in which patients can participate regardless of their hospital care setting resolves this problem. Fourth, our platform allows for real-time evaluation of response rates and data entry by participants. This allows for personalization of reminders (both the message and the timing can be adjusted) and individual guidance when participants experience difficulties in completing the questionnaire. Fifth, our platform ensures two-way communication between the participants and researchers. For example, participants were able to provide feedback on the study in an evaluation questionnaire, they were informed about publications and other relevant developments, and they were informed whether or not SARS-CoV-2-specific antibodies were detected in their blood. Lastly, our study design reduces study related costs and the burden on both participants and researchers, since visits to the local research institute are no longer necessary and participants can complete the questionnaire and perform the finger prick at home at a time that is suitable for them. Importantly, this may also make the study more environment friendly as travel movements are largely prevented.

## Challenges

We also encountered some challenges while developing and implementing our platform. First, despite automatization of data collection and processing, proper and regular correspondence with participants remains important to ensure completeness of data and to keep participants motivated for the study. With thousands of participants, it can be challenging to respond to everyone’s questions. For example, when participants received a finger prick for the first time, many of them felt the need to verify with the researchers how exactly they should perform and send in the finger prick, despite clear instructions included in the package. So when over two thousand participants received a finger prick for the first time within a time span of less than 2 weeks, we received a continuous flow of phone calls and e-mails with questions. In addition, peoples’ educational level and the extent to which they want be informed about developments of the study or in the research field in general differ considerably between participants (Table 1). Therefore, finding the right balance in the timing of sending (digital) updates and the amount (and depth) of information that is provided in the messages, while simultaneously keeping the messages understandable for everyone, is quite a challenge. Another thought-provoking aspect of a digital platform is verifying reasons for missing data. People may just forget or overlook the surveys they should complete, but when they are physically or mentally unable to complete the questionnaire, for example due to hospitalization or death, one is often dependent on correspondence of the social network of the participant. This is especially true for healthy control subjects after some time, especially in the middle of a pandemic, as they are often not registered as patients in the local research institute. Third, people may be less motivated to participate in a (fully) digitalized study due to reduced one-to-one contact time with a member of the research team. In the present study, patients and healthy controls were very motivated to participate due to the enormous impact of COVID-19 on the whole society, but it remains to be seen to what extent people are willing to participate in other research projects in the future. In our experience, giving participants something in return for their efforts, such as sharing personal or published study results, makes them feel more involved in the research project which may increase their motivation. Lastly, while data collection can be achieved without paperwork or visits to the research institute, at present, it is still mandatory to acquire written informed consent from all participants. In our view, the whole procedure of collecting and sorting informed consent forms, and then sending a copy back to the right participant requires disproportionally much time. Research platforms such as Castor have already developed electronic informed consent procedures (eConsent), so the efficiency of studies using digital data collection would considerably increase if the written informed consent forms could be replaced by eConsent.

## Potential future applications

At present, our digital research platform evolves around COVID-19 in patients with autoimmune diseases and sex- and age-matched healthy controls. However, the platform can be extended to other subjects and/or patient groups as well, especially since the finger prick test is not only suited for determining SARS-CoV-2 antibodies, but can also be used to measure most of the routine laboratory tests important for clinical care such as hemoglobin level, C-reactive protein, rheumatoid factor or other auto-antibodies, biological DMARD and anti-drug antibody levels, serum uric acid levels, and even cellular markers including immune cell subsets [6–8]. Moreover, previous studies have demonstrated satisfactory reliability of patient performed assessment of joints and disease activity score (DAS28) after only a single training [9]. Implementing patient administered physical examinations may therefore increase the applicability of digital research platforms even further, at least for studies in RA patients and perhaps even in clinical care. However, in case of required physical study visits, health-care workers who examine patients are able to directly enter the results of laboratory tests or (vital) parameters assessed during physical examinations into the platform. In the near future, the research platform will be expanded to accommodate wearable data integration, but at present, this has not been realized yet.

The two-way communication between patients and researchers implemented in our platform can be further expanded to fully take into account patients’ perspective on research. In the present study, we sent an evaluation questionnaire to participants with the aim to improve our communication and the clarity of our platform, but this can for example be expanded by asking for content related input. This would also allow for supplying tailored information to selected patient groups on new clinical trials and facilitate patient recruitment. Lastly, the platform grants possibilities for scaling up to national or even international levels. This may be especially appealing for studies in rare patient groups, when it is often challenging to obtain sufficient numbers of patients to draw firm conclusions. An example of a national (Dutch) platform that demonstrates how data mainly generated by patients can attribute to answering research questions in patients with rare diseases is the RUBRIC (Rational Use of Biologics in Rare refractory Immune-mediated inflammatory diseases (IMIDs) Consortium) registry [10, 11]. Here, patients treated with biologics and other targeted therapies score patient-reported outcome measures (PROMs) before and periodically after initiation of a new therapy via an online tool to document on their efficacy and safety. This informs physicians about new potential treatment options for patients with rare diseases, as well as treatment options that are probably not beneficial.

## Conclusion

While the COVID-19 pandemic has had detrimental effects on individuals and the society as a whole, it has also revolutionized the way in which research can be conducted. By combining blood collection via a finger prick with digital data collection, we developed a research platform in which large numbers of patients can easily be included, study visits are no longer necessary, and multicenter research, either on the national or international level, can readily be incorporated. Developing or improving already existing similar research platforms will therefore likely increase the rate, efficiency, and accuracy in which relevant research questions can be answered and may potentially also advance care settings.

## Supplementary Information

**Additional file 1.**

## Data Availability

Not applicable.

## Abbreviations

SARS-CoV-2: Severe acute respiratory syndrome coronavirus 2
DANBIO: Danish Registry for Biologic Therapies in Rheumatology
GDPR: General Data Protection Regulation
RBD: Receptor-binding domain
ELISA: Enzyme-linked immune sorbent assay
DMARD: Disease-modifying antirheumatic drugs
DAS28: Disease activity score 28
RUBRIC: Rational Use of Biologics in Rare refractory Immune-mediated inflammatory diseases Consortium
IMIDs: Immune-mediated inflammatory diseases
PROMs: Patient-reported outcome measures
