# Supplementary figures and images for: Spinning straw into gold: description of a disruptive rheumatology research platform inspired by the COVID-19 pandemic
Source: Arthritis Res Ther. 2021 Aug 5;23:207. doi: 10.1186/s13075-021-02574-z (PMC8338203; doi:10.1186/s13075-021-02574-z)

**Supplementary material**


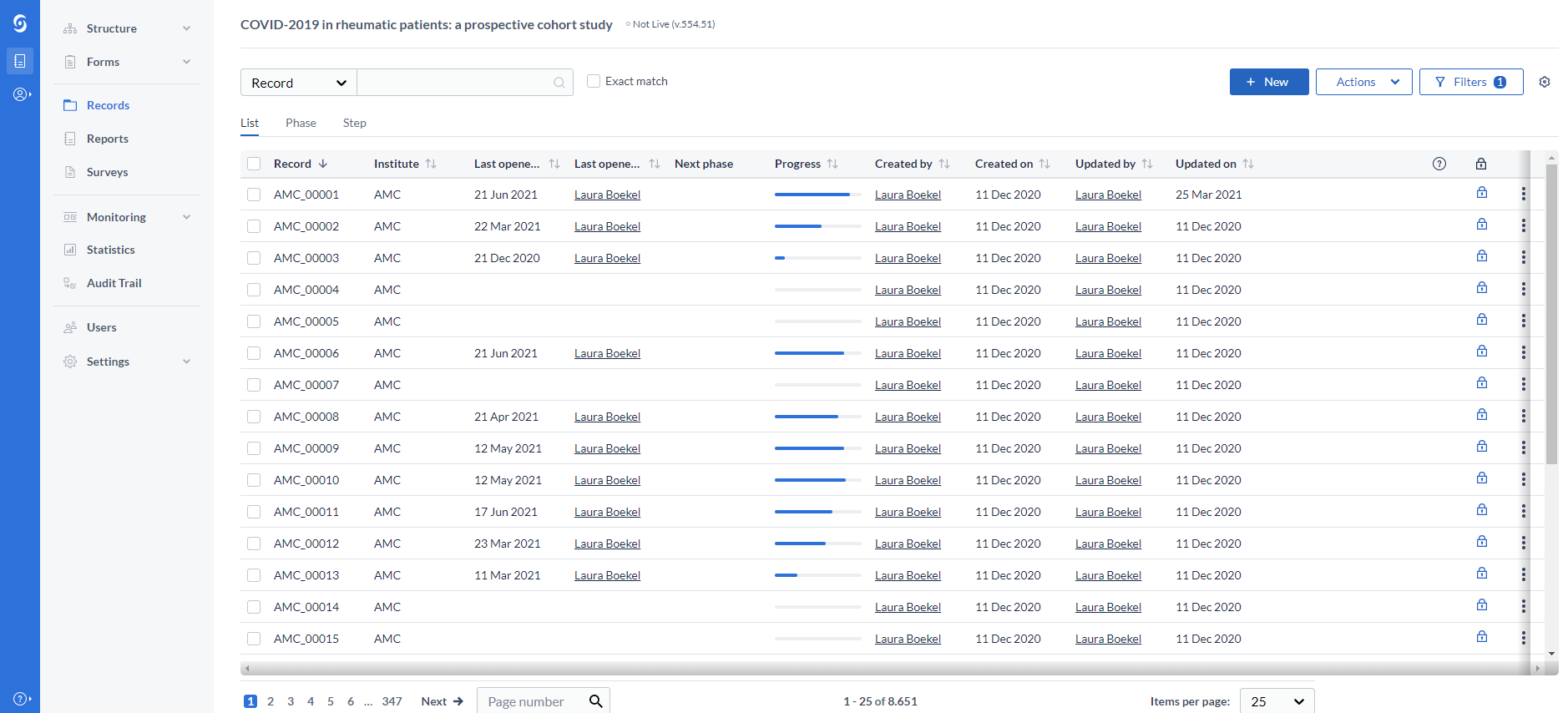
Overview of database:


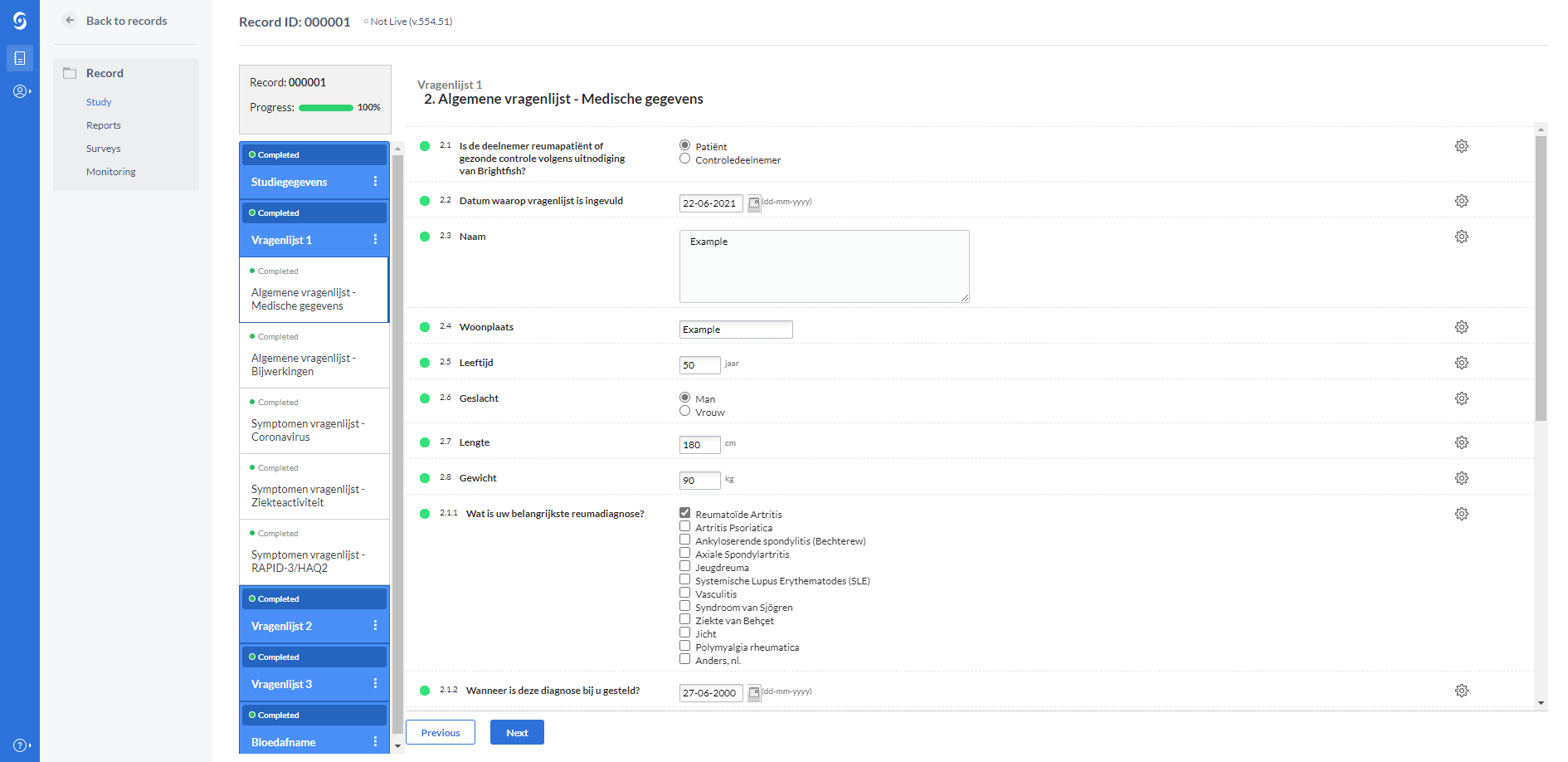
Overview of data for individual participants:

Supplement: Supplementary file 1 — Additional file 1. [file 13075_2021_2574_MOESM1_ESM.docx]
